# Supplementary material for: Psychosocial Effects and Use of Communication Technologies during Home Confinement in the First Wave of the COVID-19 Pandemic in Italy and The Netherlands
Source: Int J Environ Res Public Health. 2021 Mar 5;18(5):2619. doi: 10.3390/ijerph18052619 (PMC7967354; doi:10.3390/ijerph18052619)
Supplement: Supplementary file 1 [file ijerph-18-02619-s001.zip › full English version-Effects of Home Confinement on Multiple Lifestyle Behaviours During the COVID-19 Outbreak (ECLB-COVID19) (1).pdf]

Effects of Home Confinement on Multiple Lifestyle Behaviours During the COVID-19 Outbreak (ECLB-COVID19)

- Auswirkungen der häuslichen Isolation auf multiples Lebensstilverhalten während des COVID-19-Ausbruchs.
- Les effets du confinement à domicile sur les comportements multiples liés à nos modes de vie pendant l'épidémie de COVID-19.
- آثار الحجر الصحي على السلوكيات ونمط الحياة أثناء تفشي وباء الكورونا المستجد كوفيد-19
- تأثير خانه نشینی اجباری بر شیوه زندگی در زمان شیوع ویروس کرونا
- Efectos del Confinamiento en Múltiples Hábitos de Estilo de Vida durante el brote de COVID-19.
- Efeitos do Confinamento Doméstico em Múltiplos Comportamentos de Estilo de Vida Durante o Surto de COVID-19.
- Učinki samo-izolacije zaradi COVID-19 na življenjski slog
- Effecten van sociale isolatie maatregelen op leefstijlgedrag tijdens de uitbraak van COVID-19 (ECLB-COVID19)
- Effetti delle Misure Restrittive di Isolamento sulle Abitudini e lo Stile di Vita Durante la Pandemia COVID-19 (ECLB-COVID19)

* Required

1. Please choose your language; Bitte wählen Sie Ihre Sprache; S'il vous plaît Choisissez votre langue; Por favor elige tu idioma; Por favor escolha seu idioma; prosim, izberite svoj jezik; لطفا زبان خود را انتخاب کنید ;الرجاء اختيار لغتك *

Mark only one oval.

- English
- Deutsch *Skip to question 166*
- Français *Skip to question 89*
- اللغة العربية *Skip to question 397*
- Español *Skip to question 320*
- Português *Skip to question 243*
- Slovenščina *Skip to question 474*
- فارسی *Skip to question 551*
- Nederlands *Skip to question 719*
- Italiano *Skip to question 628*
- Greek *Skip to question 810*

Thank you for taking the time (14±2minutes) to complete this survey (6 pages). Your feedback will help us better understand changes in lifestyle behaviours during the COVID-19 confinement period.

Principal Investigator:
- Otto-von-Guericke University, Germany

Co-Investigator
- Germany: German Center for Neurodegenerative Diseases; University of Münster; Ulm University
- France: E-Senior; University of Toulouse; University Paris Nanterre; University of Paris-Est Créteil
- Tunisia: University of Sfax
- Slovenia: Science and Research Centre of Koper
- Portugal: University of Porto
- Spain: Fundesalud
- Italy: Genoa University; Catholic University of the Sacred Heart | UNICATT
- Netherland: University Twente; UMCG, Rijksuniversiteit Groningen
- Canada: University of Waterloo; York University
- UK: Loughborough University
- USA: University of Arkansas; Georgia Southern University-Armstrong Campus
- Brazil: Estácio de Sá University, Rio de Janeiro State University
- Jordan: Yarmouk University; University of Jordan
- Egypt: Assiut University
- Algeria: Djelfa University
- Qatar: Prof. Dr. Karim Chamari: Aspetar
- UAE: American University in the Emirates
- Iran: Imam Khomeini International University

Contact Person:
- Prof. Dr. Anita Hoekelmann (anita.hoekelmann@ovgu.de)
- Dr. Achraf Ammar (achraf1.ammar@ovgu.de)

Institute of Sport Science, Otto-von-Guericke University, Magdeburg, Germany

Consent of participation

During the informed consent process, survey participants were assured all data would be used only for research purposes (e.g., conferences and scientific publications) and not be disclosed or released to others without the consent of the individual. Participants' answers are anonymous and confidential according to Google's privacy policy (https://policies.google.com/privacy?hl=en). Participants don't have to mention their names or contact information. In addition, participant can stop participating in the study and can leave the questionnaire at any stage before the submission process and their responses will not be saved. Response will be saved only by clicking on "submit" button. By completing this survey, you are acknowledging the above approval form and you are consenting to voluntarily participate in this anonymous study. Please be honest in your responses.

Effects of Home Confinement on Multiple Lifestyle Behaviours During the COVID-19 Outbreak (ECLB-COVID19)	In March 2020, the World Health Organization (WHO) made the assessment that COVID-19 can be characterized as a pandemic. WHO and public health authorities around the world are acting to contain the COVID-19 outbreak through home confinement. However, this time of crisis is generating stress throughout the population.<br>The present survey aims to assess, in many countries, the effects of home confinement on multiple lifestyle behaviours during the COVID-19 outbreak. Identifying the exact changes in lifestyle behaviours during the confinement period (compared to before confinement) may help to provide efficient and effective suggestions/solutions (e.g., ICT-based solutions) to mitigate the possible unwanted psychosocial effects of home confinement.

Untitled Section

Data Privacy/Security

Data protection and privacy is of the utmost importance. The present anonymous online survey was designed only for research purpose. In our research project we give special care to data privacy and security and strives to protect the collected data against any unauthorized access by third parties with taking into consideration the latest regulations in terms of data protection.
Accordingly, Information collected in the ECLB-COVID19 is kept in strictest confidence. During the informed consent process, survey participants are assured that data collected will be used only for research purposes and will not be disclosed or released to others without the consent of the individual. However, because we are using Google Forms, participants shall also acknowledge Google's privacy policy (https://policies.google.com/privacy?hl=en).
You must be 18 or older to participate in this study.

Consent of Participation

By completing this survey, you are consenting to participate anonymously in this study.

If you are using your smartphone to answer this questionnaire, please drag the cursor to see all the possible answers

2. Country (first letter in uppercase) *

____

3. City *

____

4. Gender *

Mark only one oval.

- ( ) Male
- ( ) Female
- ( ) Other: _______________

5. Age (years old) *

____

6. Level of Education *

Mark only one oval.

- No schooling completed
- High school graduate, diploma or the equivalent
- Professional degree
- Bachelor's degree
- Master/doctorate degree

7. Marital status *

Mark only one oval.

- Single
- Married/Living as couple
- Widowed/Divorced/Separated

8. What is your current employment status? *

Check all that apply.

- [ ] Employed for wages
- [ ] Self-employed
- [ ] Out of work/Unemployed
- [ ] A student
- [ ] Retired
- [ ] Unable to work
- [ ] Problem caused by COVID-19 (e.g., leaving work, lower salary etc.)

Other: [ ] ____________________

9. What is your current state of health? *

Check all that apply.

- [ ] Healthy
- [ ] With risk factors for cardiovascular disease (e.g., high blood pressure, smoking, diabetes mellitus, obesity, high blood cholesterol, sedentarity, etc.)
- [ ] With cardiovascular disease (e.g., atherosclerosis, stroke, heart failure, heart disease, etc.)
- [ ] With cognitive impairment

10. Including yourself, how many people live in your household? *

Mark only one oval per row.

	1	2	3	4	5	more than 5
Before confinement	◯	◯	◯	◯	◯	◯
During confinement	◯	◯	◯	◯	◯	◯

11. Did you think that you are in need of psychosocial support ? *

Mark only one oval per row.

	Never	Rarely	Sometimes	Often	All times
Before confinement	◯	◯	◯	◯	◯
During confinement	◯	◯	◯	◯	◯

Short Life Satisfaction and Mental Well-being Questionnaires

Short Life Satisfaction questionnaire

12. 1. In most ways my life is close to my ideal. *

Mark only one oval per row.

	Strongly disagree	Disagree	Slightly disagree	Neither Agree Nor disagree	Slightly agree	Agree	Strongly Agree
Before confinement	◯	◯	◯	◯	◯	◯	◯
During confinement	◯	◯	◯	◯	◯	◯	◯

13. 2. So far I have gotten the important things I want in life *

Mark only one oval per row.

	Strongly disagree	Disagree	Slightly disagree	Neither Agree Nor disagree	Slightly agree	Agree	Strongly Agree
Before confinement							
During confinement							

14. 3. I am satisfied with my life. *

Mark only one oval per row.

	Strongly disagree	Disagree	Slightly disagree	Neither Agree Nor disagree	Slightly agree	Agree	Strongly Agree
Before confinement							
During confinement							

The Short Warwick-Edinburgh Mental Well-being Scale (SWEMWBS)

15. 1. I've been feeling optimistic about the future *

Mark only one oval per row.

	Never	Rarely	Sometimes	Often	All times
Before confinement					
During confinement					

16. 2. I've been feeling useful *

Mark only one oval per row.

	Never	Rarely	Sometimes	Often	All times
Before confinement					
During confinement					

17. 3. I’ve been feeling relaxed *

Mark only one oval per row.

	Never	Rarely	Sometimes	Often	All times
Before confinement					
During confinement					

18. 4. I’ve been dealing with Problems well *

Mark only one oval per row.

	Never	Rarely	Sometimes	Often	All times
Before confinement					
During confinement					

19. 5. I’ve been thinking clearly *

Mark only one oval per row.

	Never	Rarely	Sometimes	Often	All times
Before confinement					
During confinement					

20. 6. I’ve been feeling close to other people *

Mark only one oval per row.

	Never	Rarely	Sometimes	Often	All times
Before confinement					
During confinement					

21. 7. I've been able to make up my own mind about things *

Mark only one oval per row.

	Never	Rarely	Sometimes	Often	All times
Before confinement	⬭	⬭	⬭	⬭	⬭
During confinement	⬭	⬭	⬭	⬭	⬭

Social Participation Questionnary (SPQ)

22. 1. Visited family/family visit *

Mark only one oval per row.

	Never	Rarely	Sometimes	Often	All times
Before confinement	⬭	⬭	⬭	⬭	⬭
During confinement	⬭	⬭	⬭	⬭	⬭

23. 2. Visited friends or neighbours/friends or neighbours visit *

Mark only one oval per row.

	Never	Rarely	Sometimes	Often	All times
Before confinement	⬭	⬭	⬭	⬭	⬭
During confinement	⬭	⬭	⬭	⬭	⬭

24. 3. Attended church or a religious activity/group *

Mark only one oval per row.

	Never	Rarely	Sometimes	Often	All times
Before confinement	⬭	⬭	⬭	⬭	⬭
During confinement	⬭	⬭	⬭	⬭	⬭

25. 4. Used the internet/social media for communication *

Mark only one oval per row.

	Never	Rarely	Sometimes	Often	All times
Before confinement	⬭	⬭	⬭	⬭	⬭
During confinement	⬭	⬭	⬭	⬭	⬭

26. 5. Phone call for social communication *

Mark only one oval per row.

	Never	Rarely	Sometimes	Often	All times
Before confinement	⬭	⬭	⬭	⬭	⬭
During confinement	⬭	⬭	⬭	⬭	⬭

27. 6. Gone to a café/restaurant, bar or party *

Mark only one oval per row.

	Never	Rarely	Sometimes	Often	All times
Before confinement	⬭	⬭	⬭	⬭	⬭
During confinement	⬭	⬭	⬭	⬭	⬭

28. 7. Gone to the cinema or theatre or sport event *

Mark only one oval per row.

	Never	Rarely	Sometimes	Often	All times
Before confinement	⬭	⬭	⬭	⬭	⬭
During confinement	⬭	⬭	⬭	⬭	⬭

29. 8. Gone to the gym or exercise class *

Mark only one oval per row.

	Never	Rarely	Sometimes	Often	All times
Before confinement					
During confinement					

30. 9. Gone to a class *

Mark only one oval per row.

	Never	Rarely	Sometimes	Often	All times
Before confinement					
During confinement					

31. 10. Had social contact through other activities *

Mark only one oval per row.

	Never	Rarely	Sometimes	Often	All times
Before confinement					
During confinement					

32. 11. School-related group *

Mark only one oval per row.

	No	Yes
Before confinement		
During confinement		

33. 12. Volunteer organization or group *

Mark only one oval per row.

	No	Yes
Before confinement		
During confinement		

34. 13. Ethnic group *

Mark only one oval per row.

	No	Yes
Before confinement		
During confinement		

35. 14. Other group (e.g., sport, service, etc.) *

Mark only one oval per row.

	No	Yes
Before confinement		
During confinement		

Short Mood and Feelings Questionnaire (SMFQ)

36. 1. I felt miserable or unhappy *

Mark only one oval per row.

	0 (not true)	1 (sometimes)	2 (true)
Before confinement			
During confinement			

37. 2. I didn’t enjoy anything at all *

Mark only one oval per row.

	0 (not true)	1 (sometimes)	2 (true)
Before confinement	⬭	⬭	⬭
During confinement	⬭	⬭	⬭

38. 3. I felt so tired I just sat around and did nothing *

Mark only one oval per row.

	0 (not true)	1 (sometimes)	2 (true)
Before confinement	⬭	⬭	⬭
During confinement	⬭	⬭	⬭

39. 4. I was very restless *

Mark only one oval per row.

	0 (not true)	1 (sometimes)	2 (true)
Before confinement	⬭	⬭	⬭
During confinement	⬭	⬭	⬭

40. 5. I felt I was no good anymore *

Mark only one oval per row.

	0 (not true)	1 (sometimes)	2 (true)
Before confinement	⬭	⬭	⬭
During confinement	⬭	⬭	⬭

41. 6. I cried a lot *

Mark only one oval per row.

	0 (not true)	1 (sometimes)	2 (true)
Before confinement	◯	◯	◯
During confinement	◯	◯	◯

42. 7. I found it hard to think properly or concentrate *

Mark only one oval per row.

	0 (not true)	1 (sometimes)	2 (true)
Before confinement	◯	◯	◯
During confinement	◯	◯	◯

43. 8. I hated myself *

Mark only one oval per row.

	0 (not true)	1 (sometimes)	2 (true)
Before confinement	◯	◯	◯
During confinement	◯	◯	◯

44. 9. I was a bad person *

Mark only one oval per row.

	0 (not true)	1 (sometimes)	2 (true)
Before confinement	◯	◯	◯
During confinement	◯	◯	◯

45. 10. I felt lonely *

Mark only one oval per row.

	0 (not true)	1 (sometimes)	2 (true)
Before confinement	⬭	⬭	⬭
During confinement	⬭	⬭	⬭

46. 11. I thought nobody really loved me *

Mark only one oval per row.

	0 (not true)	1 (sometimes)	2 (true)
Before confinement	⬭	⬭	⬭
During confinement	⬭	⬭	⬭

47. 12. I thought I could never be as good as other people *

Mark only one oval per row.

	0 (not true)	1 (sometimes)	2 (true)
Before confinement	⬭	⬭	⬭
During confinement	⬭	⬭	⬭

48. 13. I did everything wrong *

Mark only one oval per row.

	0 (not true)	1 (sometimes)	2 (true)
Before confinement	⬭	⬭	⬭
During confinement	⬭	⬭	⬭

Physical Activity and Diet Behaviours Questionnaires

International Physical Activity Questionnaire - Short Form

49. 1. During a week, how many days did you do vigorous (hard) physical activities (e.g., lifting, digging, aerobics, or fast biking)? *

Mark only one oval per row.

	0 (Skip to question 3)	1	2	3	4	5	6	7
Before confinement	◯	◯	◯	◯	◯	◯	◯	◯
During confinement	◯	◯	◯	◯	◯	◯	◯	◯

50. 2. How much time (Minute/day) did you usually spend doing vigorous physical activities on one of those days?

(.....min) Before confinement AND (.....min) During confinement

____

51. 3. During a week, how many days did you do moderate physical activities (e.g., light loads, bicycling at a regular pace, or doubles tennis)? Do not include walking *

Mark only one oval per row.

	0 (Skip to question 5)	1	2	3	4	5	6	7
Before confinement	◯	◯	◯	◯	◯	◯	◯	◯
During confinement	◯	◯	◯	◯	◯	◯	◯	◯

52. 4. How much time did you usually spend doing moderate physical activities on one of those days?

(.....min) Before confinement AND (.....min) During confinement

____

53. 5. During a week, how many days did you do walk/recreation activity for at least 10 minutes at a time? *

Mark only one oval per row.

	0 (Skip to question 7)	1	2	3	4	5	6	7
Before confinement	⬭	⬭	⬭	⬭	⬭	⬭	⬭	⬭
During confinement	⬭	⬭	⬭	⬭	⬭	⬭	⬭	⬭

54. 6. How much time did you usually spend walking on one of those days?
(.....min) Before confinement AND (.....min) During confinement

____

55. 7. How much time did you spend sitting on a week day? *
(.....hours) Before confinement AND (.....hours) During confinement

____

56. 8. How likely are you to use internet, social media, apps , smart watch/phone, fitness tracker for physical activity purpose *

Mark only one oval per row.

	Never	Sometimes	Most of the time	Always
Before confinement	⬭	⬭	⬭	⬭
During confinement	⬭	⬭	⬭	⬭

Short Diet Behaviours Questionnaire

57. 1. How likely are you to have an unhealthy diet/food ?(high in calories from sugar or fat, colorants, salt and tropical oils; and low in fibre and vitamins (e.g., fried potato crisps/chips, cakes, white sauces) *

Mark only one oval per row.

	Never	Sometimes	Most of the time	Always
Before confinement	◯	◯	◯	◯
During confinement	◯	◯	◯	◯

58. 2. How often have you found yourself being eating out of control (binge-eating)? *

Mark only one oval per row.

	Never	Sometimes	Most of the time	Always
Before confinement	◯	◯	◯	◯
During confinement	◯	◯	◯	◯

59. 3. How many main meals do you eat a day (i.e., breakfast, lunch, dinner)? *

Mark only one oval per row.

	1-2	3	4	5	more than 5
Before confinement	◯	◯	◯	◯	◯
During confinement	◯	◯	◯	◯	◯

60. 4. How likely are you to have a snack between meals or a late night snack ? (Consider every fruit,every yoghurt, or a glass of milk etc. as a single snack) *

Mark only one oval per row.

	Never	Sometimes	Most of the time	Always
Before confinement	◯	◯	◯	◯
During confinement	◯	◯	◯	◯

61. 5. Do you engage in binge alcohol drinking? (5 or more drinks in a sitting) *

Mark only one oval per row.

	Never	Sometimes	Most of the time	Always
Before confinement	◯	◯	◯	◯
During confinement	◯	◯	◯	◯

62. 6. How likely are you to use internet, social media, apps for diet purpose (e.g., preparing meal, controling calories etc.) *

Mark only one oval per row.

	Never	Sometimes	Most of the time	Always
Before confinement	◯	◯	◯	◯
During confinement	◯	◯	◯	◯

Pittsburgh Sleep Quality Index (PSQI)

1. What time have you usually gone to bed at night?

63. 1.a. Before confinement (time to bed) *

Example: 8:30 AM

64. 1.b. During confinement (time to bed) *

Example: 8:30 AM

65. 2. How long (in minutes) has it usually taken you to fall asleep each night? *

(.....min) Before confinement AND (.....min) During confinement

3. What time have you usually gotten up in the morning?

66. 3.a. Before confinement (getten up time) *

Example: 8:30 AM

67. 3.b. During confinement (getten up time) *

Example: 8:30 AM

68. 4. How many hours of actual sleep did you get at night? (This may be different than the number of hours you spent in bed.) *

(.....hours) Before confinement AND (.....hours) During confinement

5. How often have you had trouble sleeping because you . . .

69. 5.a.Cannot get to sleep within 30 minutes *

Mark only one oval per row.

	No	Less than once a week	Once or Twice a week	Three or more times a week
Before confinement				
During confinement				

70. 5.b. Wake up in the middle of the night or early morning *

Mark only one oval per row.

	No	Less than once a week	Once or Twice a week	Three or more times a week
Before confinement				
During confinement				

71. 5.c. Have to get up to use the bathroom *

Mark only one oval per row.

	No	Less than once a week	Once or Twice a week	Three or more times a week
Before confinement	⬭	⬭	⬭	⬭
During confinement	⬭	⬭	⬭	⬭

72. 5.d. Cannot breathe comfortably, Cough or snoreloudly *

Mark only one oval per row.

	No	Less than once a week	Once or Twice a week	Three or more times a week
Before confinement	⬭	⬭	⬭	⬭
During confinement	⬭	⬭	⬭	⬭

73. 5.e. Feel too cold/hot *

Mark only one oval per row.

	No	Less than once a week	Once or Twice a week	Three or more times a week
Before confinement	⬭	⬭	⬭	⬭
During confinement	⬭	⬭	⬭	⬭

74. 5.f. Other reason (e.g., pain, use phone, bad dreams etc.) *

Mark only one oval per row.

	No	Less than once a week	Once or Twice a week	Three or more times a week
Before confinement	⬭	⬭	⬭	⬭
During confinement	⬭	⬭	⬭	⬭

75. 6. How often have you taken medicine to help you sleep (prescribed or "over the counter")? *

Mark only one oval per row.

	NO	Less than once a week	Once or Twice a week	Three or more times a week
Before confinement	⬭	⬭	⬭	⬭
During confinement	⬭	⬭	⬭	⬭

76. 7. How often have you had trouble staying awake while driving, eating meals, or engaging in social activity? *

Mark only one oval per row.

	NO	Less than once a week	Once or Twice a week	Three or more times a week
Before confinement	⬭	⬭	⬭	⬭
During confinement	⬭	⬭	⬭	⬭

77. 8. How much of a problem has it been for you to keep up enough enthusiasm to getthings done? *

Mark only one oval per row.

	No problem at all	Only a very slight problem	Somewhat of a problem	A very big problem
Before confinement	⬭	⬭	⬭	⬭
During confinement	⬭	⬭	⬭	⬭

78. 9. How would you rate your sleep quality overall? *

Mark only one oval per row.

	Very good	Fairly good	Fairly bad	Very bad
Before confinement	⬭	⬭	⬭	⬭
During confinement	⬭	⬭	⬭	⬭

Health care

79. Please select what is most applicable to you: *

Mark only one oval.

- ⬭ I currently provide unpaid care (informal care) to an elderly person (such as, e.g., a spouse, parent, other relative or neighbour).
- ⬭ I receive care from my partner/ family etc. and/ or receive professional home care. *Skip to question 86*
- ⬭ I currently provide professional care to elderly clients. *Skip to question 87*
- ⬭ None

Reason of care, housing situation, burden, and technology

80. What is the main reason of care? (multiple choices possible) *

Check all that apply.

- [ ] Aging
- [ ] Dementia or mild cognitive impairment (MCI)

Other: [ ] ______________________

81. What is your housing situation? *

Mark only one oval.

- ( ) I live together with the person I provide care for.
- ( ) The person I provide care for does not live with me together.

82. To what extent do you experience burden/ stress as a result from your care responsibilities? *

Mark only one oval per row.

	None to very small extent	Small extent	Average extent	Large extent	Very large extent
Before the COVID-19 measures	( )	( )	( )	( )	( )
During the COVID-19 measures	( )	( )	( )	( )	( )

83. Which digital communication tools do you use during the COVID-19 lockdown measures to maintain safe contact with the person you care for and how satisfied are you about these? *

Mark only one oval per row.

	Very dissatisfied	Dissatisfied	Neutral	Satisfied	Very satisfied	Not used
Telephone	( )	( )	( )	( )	( )	( )
Video calling	( )	( )	( )	( )	( )	( )
Social Media	( )	( )	( )	( )	( )	( )
Online platforms for caregivers	( )	( )	( )	( )	( )	( )
Messenger apps	( )	( )	( )	( )	( )	( )

84. Which other digital communication tool are you currently using? (If not applicable, skip this question)

____

85. How satisfied are you about this communication tool? (If not applicable, skip this question)

Mark only one oval.

	1	2	3	4	5	
Very dissatisfied	⬭	⬭	⬭	⬭	⬭	Very satisfied

Reason of care

86. What is the main reason of care? (multiple choices possible) *

Check all that apply.

- [ ] Aging
- [ ] Dementia or mild cognitive impairment (MCI)
- Other: [ ] ________________________________

Reason of care and burden

87. What is the main reason of care? (multiple choices possible) *

Check all that apply.

- [ ] Aging
- [ ] Dementia or mild cognitive impairment (MCI)
- Other: [ ] ________________________________

88. To what extent do you experience burden/ stress as a result from your care responsibilities? *

Mark only one oval per row.

	None to very small extent	Small extent	Average extent	Large extent	Very large extent
Before the COVID-19 measures	○	○	○	○	○
During the COVID-19 measures	○	○	○	○	○

Les effets du confinement à domicile sur les comportements multiples liés à nos modes de vie pendant l'épidémie de COVID-19 (ECLB-COVID19)

En mars 2020, l'Organisation mondiale de la santé (OMS) a estimé que le COVID-19 pouvait être qualifié de pandémie. L'OMS et les autorités de santé publique du monde entier agissent pour contenir l'épidémie de COVID-19 par le confinement à domicile. Cependant, cette période de crise génère un stress dans toute la population.
La présente étude vise à évaluer, dans de nombreux pays, les effets du confinement à domicile sur les comportements multiples liés au mode de vie pendant l'épidémie de COVID-19. L'identification des changements exacts dans les comportements liés au mode de vie pendant la période de confinement (par rapport à la période précédant ce confinement) peut aider à fournir des suggestions/solutions efficaces et efficientes (par exemple, des solutions basées sur les TIC) pour atténuer les éventuels effets psychosociaux indésirables du confinement à domicile.

Confidentialité et sécurité des données

La protection des données et de la vie privée est de la plus haute importance. La présente enquête anonyme en ligne a été conçue uniquement à des fins de recherche. Dans notre projet de recherche, nous accordons une attention particulière à la confidentialité et à la sécurité des données et nous nous efforçons de protéger les données collectées contre tout accès non autorisé par des tiers en tenant compte des dernières réglementations en matière de protection des données (Google's privacy policy: https://policies.google.com/privacy?hl=en)..
En conséquence, les informations recueillies dans le cadre de l'ECLB-COVID19 sont conservées dans la plus stricte confidentialité. Au cours du processus de consentement éclairé, les participants à l'enquête sont assurés que les données collectées ne seront utilisées qu'à des fins de recherche et ne seront pas divulguées ou communiquées à d'autres personnes sans le consentement de l'intéressé. Vous devez être âgé de 18 ans ou plus pour participer à cette étude.

Consentement de participation

En répondant à cette enquête, vous consentez à participer de manière anonyme à cette étude.

Si vous utilisez votre smartphone pour répondre à ce questionnaire, veuilez svp glissez le curseur afin de voir toutes les réponses possibles

89. Pays (première lettre en majuscule) *

____
