# Supplementary material for: Psychosocial Effects and Use of Communication Technologies during Home Confinement in the First Wave of the COVID-19 Pandemic in Italy and The Netherlands
Source: Int J Environ Res Public Health. 2021 Mar 5;18(5):2619. doi: 10.3390/ijerph18052619 (PMC7967354; doi:10.3390/ijerph18052619)
Supplement: Supplementary file 1 [file ijerph-18-02619-s001.zip › ITA and Dutch version-ECLB-COVID19.pdf]

Effetti delle Misure Restrittive di Isolamento sulle Abitudini e lo Stile di Vita Durante la Pandemia COVID-19 (ECLB-COVID19)

Nel Marzo 2020, l'Organizzazione Mondiale della Sanità (OMS) ha valutato che il COVID-19 è da considerarsi una pandemia. L'OMS e le autorità sanitarie nazionali stanno attuando provvedimenti per limitare la diffusione del contagio tramite il confinamento domiciliare. Tuttavia, questa situazione di crisi sta generando grande stress nella popolazione. Il presente questionario si propone di valutare l'effetto di queste misure sul comportamento e sullo stile di vita delle persone durante la pandemia di COVID-19 in diverse nazioni. Identificare i cambiamenti nello stile di vita durante il periodo di isolamento domestico (confrontandoli i dati precedenti), può aiutare nel fornire soluzioni e suggerimenti utili (es. soluzioni infomatiche) per mitigare i possibili effetti psicosociali indesiderati delle misure restrittive

Privacy/Sicurezza dei dati

La protezione e la privacy dei dati è di estrema importanza. Il presente questionario online è anonimo, ed è stato progettato a esclusivo scopo di ricerca. Nel nostro progetto di ricerca riserviamo assoluto riguardo alla privacy dei dati raccolti e ci impegnamo affinchè non siano accessibili a terze parti, tenendo in considerazione le più recenti normative di trattamento dei dati. Di conseguenza, le informazioni raccolte nella ECLB-COVID19 sono trattate con assoluta riservatezza. Nel processo di consenso informato, ai partecipanti al questionario è garantito che la raccolta dati avverrà a soli scopi di ricerca e che questi non verranno divulgati o rilasciati a terze parti senza il consenso dell'individuo. è necessario avere almeno 18 anni per partecipare allo studio.

Consenso di Partecipazione

Completando il presente questionario, acconsente a partecipare in forma anonima allo studio

If you are using your smartphone to answer this questionnaire, please drag the cursor to see all the possible answers

628. Nazionalità *

____

629. Città di residenza *

____

630. Genere *

Mark only one oval.

- Maschio
- Femmina
- Other: ______________________

631. Età *

____

632. Grado di istruzione *

Mark only one oval.

- Licenza media o inferiori
- Diploma di Scuola Superiore
- Scuola Professionale
- Laurea Triennale
- Laurea Magistrale/ Ciclo unico

633. Stato civile *

Mark only one oval.

- Single
- Sposato/ Convivente
- Divorziato/Separato/Vedovo

634. Occupazione *

Check all that apply.

- [ ] Lavoratore dipendente
- [ ] Libero Professionista
- [ ] Disocccupato
- [ ] Studente
- [ ] In pensione
- [ ] Inabile al lavoro
- [ ] Problemi causati dalla situazione COVID-19

Other: [ ] ______________________

635. Qual è il suo attuale stato di salute? *

Check all that apply.

- [ ] In salute
- [ ] A rischio per malattie cardiovascolari: (es: pressione alta, fumo di sigaretta, diabete mellito, obesità, colesterolo alto, sedentarietà ecc)
- [ ] Con malattie cardiovascolari (arterosclerosi, Ictus,Insufficienzaa cardiaca, malattie cardiache, etc)
- [ ] Con Deterioramento cognitivo

636. Di quante persone è composto il suo nucleo familiare (compreso/a lei)? *

Mark only one oval per row.

	1	2	3	4	5	Più di 5
Prima dell'isolamento	◯	◯	◯	◯	◯	◯
Durante l'isolamento	◯	◯	◯	◯	◯	◯

637. Pensa di avere bisogno di supporto psicosociale? *

Mark only one oval per row.

	Mai	Raramente	A volte	Spesso	Sempre
Prima dell'isolamento	◯	◯	◯	◯	◯
Durante l'isolamento	◯	◯	◯	◯	◯

Italian adaptation of the Short Life Satisfacttion and Mental Well-Being Questionnaires

Itaian Adaptation of The Short Life Satisfaction Questionnaire

638. 1. La mia vita è abbastanza vicina al mio ideale *

Mark only one oval per row.

	Del tutto in disaccordo	In disaccordo	Abbastanza in disaccordo	Né in accordo né in disaccordo	Abbastanza d'accordo	D'accordo	D d'…
Prima dell'isolamento	⬭	⬭	⬭	⬭	⬭	⬭	
Durante l'isolamento	⬭	⬭	⬭	⬭	⬭	⬭	

639. 2. Ho ottenuto le cose importanti che volevo nella vita *

Mark only one oval per row.

	Del tutto in disaccordo	In disaccordo	Abbastanza in disaccordo	Né in accordo né in disaccordo	Abbastanza d'accordo	D'accordo	D d'…
Prima dell'isolamento	⬭	⬭	⬭	⬭	⬭	⬭	
Durante l'isolamento	⬭	⬭	⬭	⬭	⬭	⬭	

640. 3. Sono soddisfatto della mia vita *

Mark only one oval per row.

	Del tutto in disaccordo	In disaccordo	Abbastanza in disaccordo	Né in accordo né in disaccordo	Abbastanza d'accordo	D'accordo	D d'…
Prima dell'isolamento	⬭	⬭	⬭	⬭	⬭	⬭	
Durante l'isolamento	⬭	⬭	⬭	⬭	⬭	⬭	

Versione Italiana della Scala : The Short Warwick-Edinburgh Mental Well-being Scale (SWEMWBS)

641. 1. Mi sono sentito ottimista riguardo al mio futuro *

Mark only one oval per row.

	Mai	Raramente	A volte	Spesso	Sempre
Prima dell'isolamento	◯	◯	◯	◯	◯
Durante l'isolamento	◯	◯	◯	◯	◯

642. 2. Mi sono sentito utile *

Mark only one oval per row.

	Mai	Raramente	A volte	Spesso	Sempre
Prima dell'isolamento	◯	◯	◯	◯	◯
Durante l'isolamento	◯	◯	◯	◯	◯

643. 3. Mi sono sentito rilassato *

Mark only one oval per row.

	Mai	Raramente	A volte	Spesso	Sempre
Prima dell'isolamento	◯	◯	◯	◯	◯
Durante l'isolamento	◯	◯	◯	◯	◯

644. 4. Ho affrontato bene i problemi *

Mark only one oval per row.

	Mai	Raramente	A volte	Spesso	Sempre
Prima dell'isolamento	◯	◯	◯	◯	◯
Durante l'isolamento	◯	◯	◯	◯	◯

645. 5. Ho pensato in modo chiaro *

Mark only one oval per row.

	Mai	Raramente	A volte	Spesso	Sempre
Prima dell'isolamento					
Durante l'isolamento					

646. 6. Mi sono sentito vicino ad altre persone *

Mark only one oval per row.

	Mai	Raramente	A volte	Spesso	Sempre
Prima dell'isolamento					
Durante l'isolamento					

647. 7. Sono stato in grado di prendere decisioni *

Mark only one oval per row.

	Mai	Raramente	A volte	Spesso	Sempre
Prima dell'isolamento					
Durante l'isolamento					

Adattammento Italiano della Scala: Social Participation Questionnaire (SPQ)

648. 1. Ho fatto visite ai/ricevuto visite dai miei famliari *

Mark only one oval per row.

	Mai	Raramente	A volte	Spesso	Sempre
Prima dell'isolamento					
Durante l'isolamento					

649. 2. Ho fatto visite ai/ricevuto visite dai miei vicini/ amici *

Mark only one oval per row.

	Mai	Raramente	A volte	Spesso	Sempre
Prima dell'isolamento					
Durante l'isolamento					

650. 3. Ho partecipato ad una funzione/ attività religiosa *

Mark only one oval per row.

	Mai	Raramente	A volte	Spesso	Sempre
Prima dell'isolamento					
Durante l'isolamento					

651. 4. Ho usato internet/ i social media per comunicare *

Mark only one oval per row.

	Mai	Raramente	A volte	Spesso	Sempre
Prima dell'isolamento					
Durante l'isolamento					

652. 5. Ho fatto una telefonata per comunicazioni sociali *

Mark only one oval per row.

	Mai	Raramente	A volte	Spesso	Sempre
Prima dell'isolamento					
Durante l'isolamento					

653. 6. Sono andato in un bar/ ristorante o a una festa *

Mark only one oval per row.

	Mai	Raramente	A volte	Spesso	Sempre
Prima dell'isolamento	⬭	⬭	⬭	⬭	⬭
Durante l'isolamento	⬭	⬭	⬭	⬭	⬭

654. 7. Sono andato al cinema, a teatro o ad un evento sportivo *

Mark only one oval per row.

	Mai	Raramente	A volte	Spesso	Sempre
Prima dell'isolamento	⬭	⬭	⬭	⬭	⬭
Durante l'isolamento	⬭	⬭	⬭	⬭	⬭

655. 8. Sono andato/a in palestra/ a lezione di qualche sport *

Mark only one oval per row.

	Mai	Raramente	A volte	Spesso	Sempre
Prima dell'isolamento	⬭	⬭	⬭	⬭	⬭
Durante l'isolamento	⬭	⬭	⬭	⬭	⬭

656. 9. Ho frequentato una lezione *

Mark only one oval per row.

	Mai	Raramente	A volte	Spesso	Sempre
Prima dell'isolamento	⬭	⬭	⬭	⬭	⬭
Durante l'isolamento	⬭	⬭	⬭	⬭	⬭

657. 10. Ho avuto contatti sociali tramite altre attività *

Mark only one oval per row.

	Mai	Raramente	A volte	Spesso	Sempre
Prima dell'isolamento					
Durante l'isolamento					

658. 11. Gruppi di studio *

Mark only one oval per row.

	No	Sì
Prima dell'isolamento		
Durante l'isolamento		

659. 12. Organizzazione o gruppo di volontariato *

Mark only one oval per row.

	No	Sì
Prima dell'isolamento		
Durante l'isolamento		

660. 13. Gruppo etnico *

Mark only one oval per row.

	No	Sì
Prima dell'isolamento		
Durante l'isolamento		

661. 14. Altro gruppo (es: sport, servizi etc) *

Mark only one oval per row.

	No	Sì
Prima dell'isolamento		
Durante l'isolamento		

Adattamento Italiano della Scala Short Mood and Feelings Questionnaire (SMFQ)

662. 1. Mi sono sentito depresso o infelice *

Mark only one oval per row.

	0 (non vero)	1 (a volte)	2 (vero)
Prima dell'isolamento			
Durante l'isolamento			

663. 2. Non ho goduto di niente *

Mark only one oval per row.

	0 (non vero)	1 (a volte)	2 (vero)
Prima dell'isolamento			
Durante l'isolamento			

664. 3. Mi sono sentito stanco, ho oziato e non ho fatto nulla *

Mark only one oval per row.

	0 (non vero)	1 (a volte)	2 (vero)
Prima dell'isolamento			
Durante l'isolamento			

665. 4. Ero molto inquieto *

Mark only one oval per row.

	0 (non vero)	1 (a volte)	2 (vero)
Prima dell'isolamento			
Durante l'isolamento			

666. 5. Mi sono sentito di non valere più nulla *

Mark only one oval per row.

	0 (non vero)	1 (a volte)	2 (vero)
Prima dell'isolamento			
Durante l'isolamento			

667. 6. Ho pianto molto *

Mark only one oval per row.

	0 (non vero)	1 (a volte)	2 (vero)
Prima dell'isolamento			
Durante l'isolamento			

668. 7. Ho fatto fatica a pensare con chiarezza o a concentrarmi *

Mark only one oval per row.

	0 (non vero)	1 (a volte)	2 (vero)
Prima dell'isolamento			
Durante l'isolamento			

669. 8. Ho odiato me stesso *

Mark only one oval per row.

	0 (non vero)	1 (a volte)	2 (vero)
Prima dell'isolamento	⬭	⬭	⬭
Durante l'isolamento	⬭	⬭	⬭

670. 9. Sono stato una persona molto cattiva *

Mark only one oval per row.

	0 (non vero)	1 (a volte)	2 (vero)
Prima dell'isolamento	⬭	⬭	⬭
Durante l'isolamento	⬭	⬭	⬭

671. 10. Mi sono sentito solo *

Mark only one oval per row.

	0 (non vero)	1 (a volte)	2 (vero)
Prima dell'isolamento	⬭	⬭	⬭
Durante l'isolamento	⬭	⬭	⬭

672. 11. Ho pensato che nessuno mi amasse davvero *

Mark only one oval per row.

	0 (non vero)	1 (a volte)	2 (vero)
Prima dell'isolamento	⬭	⬭	⬭
Durante l'isolamento	⬭	⬭	⬭

673. 12. Ho pensato di non poter mai essere all'altezza degli altri *

Mark only one oval per row.

	0 (non vero)	1 (a volte)	2 (vero)
Prima dell'isolamento			
Durante l'isolamento			

674. 13. Ho sbagliato tutto *

Mark only one oval per row.

	0 (non vero)	1 (a volte)	2 (vero)
Prima dell'isolamento			
Durante l'isolamento			

Questionario internazionale sull'attività fisica (IPAQ italiano)

Questionario internazionale sull'attività fisica (IPAQ italiano)

675. 1. Su sette giorni, quanti giorni hai svolto attività fisica vigorosa? (Es: sollevare pesi, scavare, aerobica, pedalare veloce) *

Mark only one oval per row.

	0 (Passare a domanda 3)	1	2	3	4	5	6	7
Prima dell'isolamento								
Durante l'isolamento								

676. 2. Quanto tempo in totale di solito trascorri in attività fisiche vigorose in uno di quei giorni?

(...minuti) prima dell'isolamento E (....minuti) durante l'isolamento

677. 3. Su sette giorni, in quanti giorni hai svolto attività fisica moderata? (non includa "camminare) *

Mark only one oval per row.

	0 (Passare a domanda 3)	1	2	3	4	5	6	7
Prima dell'isolamento								
Durante l'isolamento								

678. 4. Quanto tempo in totale di solito trascorri in attività fisiche moderate in uno di quei giorni?

(...minuti) prima dell'isolamento E (….minuti) durante l'isolamento

679. 5. Durante sette giorni, in quanti giorni hai camminato/ fatto attività fisica moderata? *

Mark only one oval per row.

	0 (Passare a domanda 3)	1	2	3	4	5	6	7
Prima dell'isolamento								
Durante l'isolamento								

680. 6. Quanto tempo in totale trascorri camminando in uno di questi giorni?

(...minuti) prima dell'isolamento E (….minuti) durante l'isolamento

681. 7. Durante gli ultimi sette giorni, in un giorno della settimana, quanto tempo hai trascorso stando seduto? *

(...ore) prima dell'isolamento E (....ore) durante l'isolamento

682. 8. Quanto è probabile che usi internet, i social media, le app, lo smartphone/smartwatch, fitness tracker per scopi riguardanti l'allenamento fisico? *

Mark only one oval per row.

	Mai	A volte	Spesso	Sempre
Prima dell'isolamento				
Durante l'isolamento				

Adattamento Italiano della Scala: Short Diet Behavior Questionnaire

683. 1. Quanto spesso mangia cibo non salutare? (Altamente calorico, con molti zuccheri, grassi, sale, oli vegetali, a basso contenuto di fibre e vitamine? Es: Patatine fritte, torte, salse) *

Mark only one oval per row.

	Mai	A volte	Spesso	Sempre
Prima dell'isolamento				
Durante l'isolamento				

684. 2. Quante volte le è capitato di mangiare senza controllo? (Binge-eating/ abbuffate) *

Mark only one oval per row.

	Mai	A volte	Spesso	Sempre
Prima dell'isolamento				
Durante l'isolamento				

685. 3. Quanti pasti completi consuma al giorno (es. colazione, pranzo, cena) *

Mark only one oval per row.

	1-2	3	4	5	Più di 5
Prima dell'isolamento	◯	◯	◯	◯	◯
Durante l'isolamento	◯	◯	◯	◯	◯

686. 4. Quanto spesso fa uno spuntino tra i pasti o a tarda sera? *

Mark only one oval per row.

	Mai	A volte	Spesso	Sempre
Prima dell'isolamento	◯	◯	◯	◯
Durante l'isolamento	◯	◯	◯	◯

687. 5. Consuma quantità eccessive di alcool? (più di 5 bicchieri in una volta) *

Mark only one oval per row.

	Mai	A volte	Spesso	Sempre
Prima dell'isolamento	◯	◯	◯	◯
Durante l'isolamento	◯	◯	◯	◯

688. 6. Quanto spesso usa internet, i social media, le app a scopi che riguardino la dieta? (es. preparare i pasti, conteggiare le calorie etc) *

Mark only one oval per row.

	Mai	A volte	Spesso	Sempre
Prima dell'isolamento	◯	◯	◯	◯
Durante l'isolamento	◯	◯	◯	◯

PSQI Italian Version

1. Di solito a che ora va a letto la sera

689. 1a. Prima dell'isolamento (a che ora) *

Example: 8:30 AM

690. 1b. Dopo l'isolamento (a che ora) *

Example: 8:30 AM

691. 2. Di solito, quanto tempo (in minuti) impiega ad addormentarsi ogni notte? *
(...minuti) prima dell'isolamento E (….minuti) durante l'isolamento

3. Quante ore ha dormito effettivamente per notte? (Potrebbero essere diverse dal numero di ore passate a letto)

692. 3a. Prima dell'isolamento (a che ora si è alzato) *

Example: 8:30 AM

693. 3b. Dopo l'isolamento (a che ora si è alzato) *

Example: 8:30 AM

694. 4. Quante ore ha dormito effettivamente per notte? (potrebbero essere diverse dal numero di ore passate a letto) *
(…ore) prima dell'isolamento E (….ore) durante l'isolamento

5. Quanto spesso ha avuto problemi di sonno dovuti a...

695. 5.a Non riuscire ad addormentarsi entro 30 minuti *

Mark only one oval per row.

	No	Meno di una volta a settimana	Una o due volte a settimana	Tre o più votle a settimana
Prima dell'isolamento				
Durante l'isolamento				

696. 5.b. Svegliarsi nel mezzo della notte o al mattino presto senza riaddormentarsi *

Mark only one oval per row.

	No	Meno di una volta a settimana	Una o due volte a settimana	Tre o più votle a settimana
Prima dell'isolamento				
Durante l'isolamento				

697. 5.c.Alzarsi nel mezzo della notte per andare in bagno *

Mark only one oval per row.

	No	Meno di una volta a settimana	Una o due volte a settimana	Tre o più votle a settimana
Prima dell'isolamento				
Durante l'isolamento				

698. 5.d. Non riuscire a respirare bene *

Mark only one oval per row.

	No	Meno di una volta a settimana	Una o due volte a settimana	Tre o più votle a settimana
Prima dell'isolamento	⬭	⬭	⬭	⬭
Durante l'isolamento	⬭	⬭	⬭	⬭

699. 5.e. Sentire troppo caldo/freddo *

Mark only one oval per row.

	No	Meno di una volta a settimana	Una o due volte a settimana	Tre o più votle a settimana
Prima dell'isolamento	⬭	⬭	⬭	⬭
Durante l'isolamento	⬭	⬭	⬭	⬭

700. 5.f. Altro (es: dolori, uso del telefono, brutti sogni etc) *

Mark only one oval per row.

	No	Meno di una volta a settimana	Una o due volte a settimana	Tre o più votle a settimana
Prima dell'isolamento	⬭	⬭	⬭	⬭
Durante l'isolamento	⬭	⬭	⬭	⬭

701. 6. Quanto spesso ha preso farmaci (prescritti dal medico o meno) per aiuitarsi a dormire? *

Mark only one oval per row.

	No	Meno di una volta a settimana	Una o due volte a settimana	Tre o più votle a settimana
Prima dell'isolamento				
Durante l'isolamento				

702. 7. Quanto spesso ha avuto difficoltà a rimanere sveglia/o alla guida o nel corso di attività sociali *

Mark only one oval per row.

	No	Meno di una volta a settimana	Una o due volte a settimana	Tre o più votle a settimana
Prima dell'isolamento				
Durante l'isolamento				

703. 8. Ha avuto problemi ad avere energie sufficienti per concludere le sue normali attività? *

Mark only one oval per row.

	Nessun problema	Piccoli problemi	Qualche problema	Grandi problemi
Prima dell'isolamento				
Durante l'isolamento				

704. 9. Come valuta complessivamente la sua qualità del sonno? *

Mark only one oval per row.

	Molto buona	Abbastanza buona	Non molto buona	Pessima
Prima dell'isolamento	⬭	⬭	⬭	⬭
Durante l'isolamento	⬭	⬭	⬭	⬭

Domande Aggiuntive

705. Indichi qual è la sua condizione attuale *

Mark only one oval.

- ⬭ Al momento, presto assistenza domiciliare gratuita (caregiver informale) ad una persona anziana (es. Coniugi, genitori, parenti, vicini)
- ⬭ Un membro della mia famiglia (o un coniuge, un vicino o altri) mi presta assistenza domiciliare gratuitamente *Skip to question 709*
- ⬭ Al momento, presto assistenza domiciliare professionale ad una persona anziana *Skip to question 710*
- ⬭ Nessuna delle precedenti *Skip to question 713*

Ragione della cura, situazione abitativa e onere della cura

706. Qual è la ragione del bisogno di assistenza? (è possible selezionare più di una risposta) *

Check all that apply.

- ☐ Età avanzata
- ☐ Demenza o deterioramento cognitivo

Other: ☐ ____________________

707. Qual è la sua condizione abitativa? *

Mark only one oval.

- ⬭ Abito con la persona di cui mi prendo cura
- ⬭ Non abito con la persona di cui mi prendo cura

708. Quanto l'assistenza che presta grava sul suo senso di responsabilità/ sul suo livello di stress? *

Mark only one oval per row.

	Per nulla	Poco	Mediamente	Molto	Moltissimo
Prima delle misure restrittive dovute al COVID-19	◯	◯	◯	◯	◯
Durante le misure restrittive dovute al COVID-19	◯	◯	◯	◯	◯

Skip to question 713

Ragione della cura

709. Qual è la ragione del bisogno di assistenza? *

Check all that apply.

- [ ] Età avanzata
- [ ] Demenza o deterioramento cognitivo

Other: [ ] ____________________

Skip to question 713

Ragione e onere della cura

710. Indichi quali delle seguenti opzioni è applicabile alla sua condizione attuale *

Mark only one oval.

◯ Al momento, presto assistenza domiciliare professionale ad una persona anziana

◯ Al momento, presto assistenza professionale ad una persona anziana presso una casa di cura (RSA)

711. Qual è la ragione del bisogno di assistenza? *

Check all that apply.

- [ ] Età avanzata
- [ ] Demenza o deterioramento cognitivo

Other: [ ] ____________________

712. Quanto l'assistenza che presta grava sul suo senso di responsabilità/ sul suo livello di stress? *

Mark only one oval per row.

	Per nulla	Poco	Mediamente	Molto	Moltissimo
Prima delle misure restrittive dovute al COVID-19					
Durante le misure restrittive dovute al COVID-19					

Skip to question 713

Solitudine

713. Quanto si sente solo/a? *

Mark only one oval per row.

	Per nulla	Poco	Mediamente	Molto	Moltissimo
Prima delle misure restrittive dovute al COVID-19					
Durante le misure restrittive dovute al COVID-19					

Uso di tecnologie di comunicazione

714. Quali dei seguenti mezzi di comunicazione utilizzava PRIMA dell'isolamento per rimanere in contatto con gli altri e quanto si riteneva soddisfatto di questi? Se presta assistenza domiciliare gratuita ad un anziano, faccia riferimento agli strumenti che le consentono di rimanere in contatto con lui/lei *

Mark only one oval per row.

	Del tutto insoddisfatto	Insoddisfatto	Neutro	Soddisfatto	Molto soddisfatto	Non lo usavo
Telefono	◯	◯	◯	◯	◯	◯
Videochiamate	◯	◯	◯	◯	◯	◯
Social Media	◯	◯	◯	◯	◯	◯
Piattaforme online per caregivers	◯	◯	◯	◯	◯	◯
App di messaggistica istantanea (es. Whatsapp)	◯	◯	◯	◯	◯	◯

715. Quali dei seguenti mezzi di comunicazione utilizza ORA per rimanere in contatto con gli altri e quanto ne è soddisfatto? Se si prende cura gratuitamente di un anziano, faccia riferimento agli strumenti che le consentono di rimanere in contatto con lui/lei *

Mark only one oval per row.

	Del tutto insoddisfatto	Insoddisfatto	Neutro	Soddisfatto	Del tutto soddisfatto	Non lo usavo
Telefono	◯	◯	◯	◯	◯	◯
Videochiamata	◯	◯	◯	◯	◯	◯
Social Media	◯	◯	◯	◯	◯	◯
Piattaforme online per caregivers	◯	◯	◯	◯	◯	◯
App di messaggistica istantanea (es. Whatsapp)	◯	◯	◯	◯	◯	◯

716. Quali altri mezzi di comunicazione sta utilizzando? (Se non applicabile, salti questa domanda)

717. Quanto è soddisfatto di questi mezzi? (Se non applicabile, salti questa domanda)

Mark only one oval.

	1	2	3	4	5	
Molto insoddisfatto	⬭	⬭	⬭	⬭	⬭	Molto soddisfatto

718. Pensiamo che sia molto importante tener conto della Sua opinione nello sviluppo e miglioria delle tecnologie che offrono supporto. Sarebbe interessato ad essere ricontattato per ricerche future? In caso affermativo, può indicare il suo indirizzo email (volontariamente)

"Effecten van sociale isolatie maatregelen op leefstijlgedrag tijdens de uitbraak van COVID-19 (ECLB-COVID19) "

In maart 2020 heeft de Wereldgezondheidsorganisatie (WHO) COVID-19 als een pandemie geclassificeerd. De WHO en volksgezondheidsautoriteiten over de hele wereld ondernemen actie om de uitbraak van COVID-19 te beperken door middel van fysiek contact te minimaliseren en zoveel mogelijk thuis te blijven. Deze crisissituatie zorgt voor stress onder de bevolking. Het identificeren van de exacte veranderingen in leefstijlgedrag tijdens de pandemie maatregelen (in vergelijking met vóór de pandemie maatregelen) kan helpen om effectieve oplossingen te vinden (bijv. ICT-gebaseerde oplossingen) om de mogelijke ongewenste psychosociale effecten van sociale isolatie te verzachten.

Privacy/ gegevensbescherming

Gegevensbescherming is van het allergrootste belang. Deze anonieme online enquête is uitsluitend ontworpen voor onderzoeksdoeleinden. Wij besteden in dit onderzoek speciale aandacht aan privacy en beschermen de verzamelde gegevens tegen toegang door derden, rekening houdend met de nieuwste regelgeving op het gebied van gegevensbescherming. De verzamelde informatie wordt strikt vertrouwelijk gehouden, uitsluitend gebruikt voor onderzoeksdoeleinden en zal nooit worden vrijgegeven aan anderen zonder toestemming van de participant. Om deel te nemen aan deze studie moet u 18 jaar of ouder zijn.

Toestemming voor deelname

Door deze enquête in te vullen geeft u toestemming om anoniem deel te nemen aan deze studie.

If you are using your smartphone to answer this questionnaire, please drag the cursor to see all the possible answers

719. Land (eerste letter in hoofdletters) *

____

720. Stad *

____

721. Geslacht *

Mark only one oval.

- ( ) Man
- ( ) Vrouw
- ( ) Other: _______________

722. Leeftijd (in jaren) *

____

723. Opleidingsniveau *

Mark only one oval.

- ( ) Geen scholing voltooid of basisonderwijs
- ( ) Voortgezet onderwijs (VMBO, HAVO, VWO)
- ( ) Middelbaar beroepsonderwijs (MBO)
- ( ) Bachelor diploma: Hoger beroepsonderwijs (HBO) of wetenschappelijk onderwijs (WO)
- ( ) Master diploma: WO of doctoraal

724. Burgerlijke staat *

Mark only one oval.

- Alleenstaand
- Gehuwd/ partnerschap
- Weduwe/wedenaar/gescheiden

725. Wat is uw huidige arbeidsstatus? *

Check all that apply.

- In loondienst
- Zelfstandig
- Werkloos
- Student
- Gepensioneerd
- Niet in staat om te werken
- Probleem door COVID-19 situatie (bijv. uit dienst getreden, lager salaris etc.)

Other: ______________________

726. Wat is uw huidige gezondheidstoestand? *

Check all that apply.

- Gezond
- Met risicofactoren voor hart- en vaatziekten (bijv. hoge bloeddruk, roken, diabetes, obesitas, hoog cholesterolgehalte in het bloed, gebrek aan beweging)
- Met hart- en vaatziekte (bijv. Atherosclerose, beroerte, hartfalen, hartaandoening etc.)
- Met cognitieve beperking

727. Uzelf inbegrepen, hoeveel mensen wonen er in uw huishouden? *

Mark only one oval per row.

	1	2	3	4	5	Meer dan 5
Vóór sociale isolatie						
Tijdens sociale isolatie						

728. Denkt u dat u psychosociale ondersteuning nodig heeft? *

Mark only one oval per row.

	Nooit	Zelden	Soms	Vaak	Altijd
Vóór sociale isolatie	⬭	⬭	⬭	⬭	⬭
Tijdens sociale isolatie	⬭	⬭	⬭	⬭	⬭

Vragen over levenstevredenheid en mentaal welbevinden

Korte vragenlijst levenstevredenheid

729. 1. In de meeste opzichten komt mijn leven dicht bij mijn ideaal. *

Mark only one oval per row.

	Helemaal oneens	Oneens	Beetje oneens	Niet eens, niet oneens	Beetje eens	Eens	Helemaal eens
Vóór sociale isolatie	⬭	⬭	⬭	⬭	⬭	⬭	⬭
Tijdens sociale isolatie	⬭	⬭	⬭	⬭	⬭	⬭	⬭

730. 2. Tot dusver heb ik de belangrijkste dingen die ik graag wilde in mijn leven gekregen. *

Mark only one oval per row.

	Helemaal oneens	Oneens	Beetje oneens	Niet eens, niet oneens	Beetje eens	Eens	Helemaal eens
Vóór sociale isolatie	⬭	⬭	⬭	⬭	⬭	⬭	⬭
Tijdens sociale isolatie	⬭	⬭	⬭	⬭	⬭	⬭	⬭

731. 3. Ik ben tevreden met mijn leven. *

Mark only one oval per row.

	Helemaal oneens	Oneens	Beetje oneens	Niet eens, niet oneens	Beetje eens	Eens	Helemaal eens
Vóór sociale isolatie							
Tijdens sociale isolatie							

Korte vragenlijst mentaal welbevinden

732. 1. Ik was optimistisch over de toekomst. *

Mark only one oval per row.

	Nooit	Zelden	Soms	Vaak	Altijd
Vóór sociale isolatie					
Tijdens sociale isolatie					

733. 2. Ik voelde me nuttig. *

Mark only one oval per row.

	Nooit	Zelden	Soms	Vaak	Altijd
Vóór sociale isolatie					
Tijdens sociale isolatie					

734. 3. Ik voelde me ontspannen. *

Mark only one oval per row.

	Nooit	Zelden	Soms	Vaak	Altijd
Vóór sociale isolatie					
Tijdens sociale isolatie					

735. 4. Ik kon goed omgaan met problemen. *

Mark only one oval per row.

	Nooit	Zelden	Soms	Vaak	Altijd
Vóór sociale isolatie	◯	◯	◯	◯	◯
Tijdens sociale isolatie	◯	◯	◯	◯	◯

736. 5. Ik kon helder denken. *

Mark only one oval per row.

	Nooit	Zelden	Soms	Vaak	Altijd
Vóór sociale isolatie	◯	◯	◯	◯	◯
Tijdens sociale isolatie	◯	◯	◯	◯	◯

737. 6. Ik voelde een hechte band met andere mensen. *

Mark only one oval per row.

	Nooit	Zelden	Soms	Vaak	Altijd
Vóór sociale isolatie	◯	◯	◯	◯	◯
Tijdens sociale isolatie	◯	◯	◯	◯	◯

738. 7. Ik kon zelfstandig beslissingen nemen. *

Mark only one oval per row.

	Nooit	Zelden	Soms	Vaak	Altijd
Vóór sociale isolatie	◯	◯	◯	◯	◯
Tijdens sociale isolatie	◯	◯	◯	◯	◯

Vragenlijst sociale participatie

739. 1. Familie bezocht/ bezoek van familie *

Mark only one oval per row.

	Nooit	Zelden	Soms	Vaak	Altijd
Vóór sociale isolatie					
Tijdens sociale isolatie					

740. 2. Vrienden/ buren bezocht/ bezoek van vrienden of buren *

Mark only one oval per row.

	Nooit	Zelden	Soms	Vaak	Altijd
Vóór sociale isolatie					
Tijdens sociale isolatie					

741. 3. Bezocht kerk of religieuze activiteit/ groep *

Mark only one oval per row.

	Nooit	Zelden	Soms	Vaak	Altijd
Vóór sociale isolatie					
Tijdens sociale isolatie					

742. 4. Gebruikte internet/ social media voor communicatie *

Mark only one oval per row.

	Nooit	Zelden	Soms	Vaak	Altijd
Vóór sociale isolatie					
Tijdens sociale isolatie					

743. 5. Telefoneerde voor sociale communicatie *

Mark only one oval per row.

	Nooit	Zelden	Soms	Vaak	Altijd
Vóór sociale isolatie					
Tijdens sociale isolatie					

744. 6. Bezocht café/ restaurant, bar of feest *

Mark only one oval per row.

	Nooit	Zelden	Soms	Vaak	Altijd
Vóór sociale isolatie					
Tijdens sociale isolatie					

745. 7. Bezocht bioscoop, theater of sportevenement *

Mark only one oval per row.

	Nooit	Zelden	Soms	Vaak	Altijd
Vóór sociale isolatie					
Tijdens sociale isolatie					

746. 8. Bezocht de sportschool of een sport cursus *

Mark only one oval per row.

	Nooit	Zelden	Soms	Vaak	Altijd
Vóór sociale isolatie					
Tijdens sociale isolatie					

747. 9. Een les gevolgd *

Mark only one oval per row.

	Nooit	Zelden	Soms	Vaak	Altijd
Vóór sociale isolatie					
Tijdens sociale isolatie					

748. 10. Had sociaal contact via andere activiteiten *

Mark only one oval per row.

	Nooit	Zelden	Soms	Vaak	Altijd
Vóór sociale isolatie					
Tijdens sociale isolatie					

749. 11. School-gerelateerde groep *

Mark only one oval per row.

	Nee	Ja
Vóór sociale isolatie		
Tijdens sociale isolatie		

750. 12. Vrijwilligersorganisatie of -groep *

Mark only one oval per row.

	Nee	Ja
Vóór sociale isolatie		
Tijdens sociale isolatie		

751. 13. Minderheden groep *

Mark only one oval per row.

	Nee	Ja
Vóór sociale isolatie	◯	◯
Tijdens sociale isolatie	◯	◯

752. 14. Andere groep (bijv. sport, dienstverlening etc.) *

Mark only one oval per row.

	Nee	Ja
Vóór sociale isolatie	◯	◯
Tijdens sociale isolatie	◯	◯

Vragenlijst over stemming en gevoelens

753. 1. Ik voelde me ellendig of ongelukkig. *

Mark only one oval per row.

	0 (niet waar)	1 (soms)	2 (waar)
Vóór sociale isolatie	◯	◯	◯
Tijdens sociale isolatie	◯	◯	◯

754. 2. Ik genoot helemaal nergens van. *

Mark only one oval per row.

	0 (niet waar)	1 (soms)	2 (waar)
Vóór sociale isolatie	◯	◯	◯
Tijdens sociale isolatie	◯	◯	◯

755. 3. Ik voelde me zo moe dat ik rond hing en niets deed. *

Mark only one oval per row.

	0 (niet waar)	1 (soms)	2 (waar)
Vóór sociale isolatie			
Tijdens sociale isolatie			

756. 4. Ik was erg rusteloos. *

Mark only one oval per row.

	0 (niet waar)	1 (soms)	2 (waar)
Vóór sociale isolatie			
Tijdens sociale isolatie			

757. 5. Ik voelde mij nutteloos. *

Mark only one oval per row.

	0 (niet waar)	1 (soms)	2 (waar)
Vóór sociale isolatie			
Tijdens sociale isolatie			

758. 6. Ik huilde veel. *

Mark only one oval per row.

	0 (niet waar)	1 (soms)	2 (waar)
Vóór sociale isolatie			
Tijdens sociale isolatie			

759. 7. Ik vond het moeilijk om helder na te denken of me te concentreren. *

Mark only one oval per row.

	0 (niet waar)	1 (soms)	2 (waar)
Vóór sociale isolatie			
Tijdens sociale isolatie			

760. 8. Ik haatte mezelf. *

Mark only one oval per row.

	0 (niet waar)	1 (soms)	2 (waar)
Vóór sociale isolatie			
Tijdens sociale isolatie			

761. 9. Ik was een slecht persoon. *

Mark only one oval per row.

	0 (niet waar)	1 (soms)	2 (waar)
Vóór sociale isolatie			
Tijdens sociale isolatie			

762. 10. Ik voelde me eenzaam. *

Mark only one oval per row.

	0 (niet waar)	1 (soms)	2 (waar)
Vóór sociale isolatie			
Tijdens sociale isolatie			

763. 11. Ik dacht dat niemand echt van me hield. *

Mark only one oval per row.

	0 (niet waar)	1 (soms)	2 (waar)
Vóór sociale isolatie			
Tijdens sociale isolatie			

764. 12. Ik dacht dat ik nooit zo goed zou zijn als andere mensen. *

Mark only one oval per row.

	0 (niet waar)	1 (soms)	2 (waar)
Vóór sociale isolatie			
Tijdens sociale isolatie			

765. 13. Ik deed alles verkeerd. *

Mark only one oval per row.

	0 (niet waar)	1 (soms)	2 (waar)
Vóór sociale isolatie			
Tijdens sociale isolatie			

Vragenlijsten over beweeg- en eetgedrag

Korte vragenlijst beweeggedrag

766. 1. Op hoeveel dagen per week heeft u zware fysieke activiteiten gedaan (bijv. aerobics, lopen of snel fietsen)? *

Mark only one oval per row.

	0 (verder met vraag 3)	1	2	3	4	5	6	7
Vóór sociale isolatie	⬭	⬭	⬭	⬭	⬭	⬭	⬭	⬭
Tijdens sociale isolatie	⬭	⬭	⬭	⬭	⬭	⬭	⬭	⬭

767. 2. Hoeveel tijd (in minuten) heeft u op zo'n dag besteedt aan zware fysieke activiteiten?

(...min) vóór sociale isolatie EN (...min) tijdens socialte isolatie

____

768. 3. Op hoeveel dagen per week heeft u matige fysieke activiteiten gedaan (bijv. fietsen aan een middelmatig tempo, zwemmen aan een middelmatig tempo of tennis dubbelspel)? (Wandelen niet inbegrepen) *

Mark only one oval per row.

	0 (verder met vraag 3)	1	2	3	4	5	6	7
Vóór sociale isolatie	⬭	⬭	⬭	⬭	⬭	⬭	⬭	⬭
Tijdens sociale isolatie	⬭	⬭	⬭	⬭	⬭	⬭	⬭	⬭

769. 4. Hoeveel tijd heeft u op zo'n dag besteedt aan matige fysieke activiteiten?

(...min) vóór sociale isolatie EN (...min) tijdens socialte isolatie

____

770. 5. Op hoeveel dagen per week heeft u gewandeld gedurende minstens 10 minuten aan één stuk? *

Mark only one oval per row.

	0 (verder met vraag 3)	1	2	3	4	5	6	7
Vóór sociale isolatie	⬭	⬭	⬭	⬭	⬭	⬭	⬭	⬭
Tijdens sociale isolatie	⬭	⬭	⬭	⬭	⬭	⬭	⬭	⬭

771. 6. Hoeveel tijd heeft u op zo´n dag gewandeld?

(...min) vóór sociale isolatie EN (...min) tijdens socialte isolatie

____

772. 7. Hoeveel tijd heeft u gemiddeld gezeten op een weekdag? *

(...uren) vóór sociale isolatie EN (...uren) tijdens socialte isolatie

____

773. 8. Hoe waarschijnlijk acht u het dat u internet, social media, apps, smart watch/phone of een fitness tracker gebruikt voor fysieke activiteiten? *

Mark only one oval per row.

	Nooit	Soms	Meestal	Altijd
Vóór sociale isolatie	⬭	⬭	⬭	⬭
Tijdens sociale isolatie	⬭	⬭	⬭	⬭

Korte vragenlijst over uw eetpatroon

774. 1. Hoe waarschijnlijk is het dat u ongezond eet? (rijk aan calorieën uit suiker of vet, kleurstoffen, zout en tropische olieën; weinig vezels en vitamines, bijv. gefrituurde aardappelen, cakes, witte sauzen)? *

Mark only one oval per row.

	Nooit	Soms	Meestal	Altijd
Vóór sociale isolatie	⬭	⬭	⬭	⬭
Tijdens sociale isolatie	⬭	⬭	⬭	⬭

775. 2. Hoe vaak heeft u gemerkt dat uw eetgedrag uit de hand loopt (eetbuien)? *

Mark only one oval per row.

	Nooit	Soms	Meestal	Altijd
Vóór sociale isolatie	⬭	⬭	⬭	⬭
Tijdens sociale isolatie	⬭	⬭	⬭	⬭

776. 3. Hoeveel hoofdmaaltijden nuttigt u dagelijks (bijv. ontbijt, lunch, diner)? *

Mark only one oval per row.

	1 of 2	3	4	5	Meer dan 5
Vóór sociale isolatie	⬭	⬭	⬭	⬭	⬭
Tijdens sociale isolatie	⬭	⬭	⬭	⬭	⬭

777. 4. Hoe waarschijnlijk acht u het dat u een snack neemt tussen de maaltijden door of 's avonds laat? (Beschouw elk stuk fruit, elke portie yoghurt of glas melk etc. als een enkele snack.) *

Mark only one oval per row.

	Nooit	Soms	Meestal	Altijd
Vóór sociale isolatie	⬭	⬭	⬭	⬭
Tijdens sociale isolatie	⬭	⬭	⬭	⬭

778. 5. Drinkt u 5 of meer glazen alcohol tijdens 1 gelegenheid (binge drinken)? *

Mark only one oval per row.

	Nooit	Soms	Meestal	Altijd
Vóór sociale isolatie				
Tijdens sociale isolatie				

779. 6. Hoe waarschijnlijk acht u het dat u internet, social media of apps gebruikt voor dieetdoeleinden (bijv. maaltijd bereiden, beheersen van calorieën etc.)? *

Mark only one oval per row.

	Nooit	Soms	Meestal	Altijd
Vóór sociale isolatie				
Tijdens sociale isolatie				

Vragenlijst slaapkwaliteit

1. Om hoe laat bent u ´s avonds gewoonlijk naar bed gegaan?

780. 1a. Vóór sociale isolatie (bedtijd) *

Example: 8:30 AM

781. 1b. Tijdens sociale isolatie (bedtijd) *

Example: 8:30 AM

782. 2. Hoeveel tijd (in minuten) had u gewoonlijk nodig om ´s avonds in slaap te vallen? *

(...min) vóór sociale isolatie EN (...min) tijdens sociale isolatie

3. Om hoe laat bent u gewoonlijk ´s ochtends opgestaan?

783. 3a. Vóór sociale isolatie (tijdstip van opstaan) *

Example: 8:30 AM

784. 3b. Tijdens sociale isolatie (tijdstip van opstaan) *

Example: 8:30 AM

785. 4. Hoveel uren slaap hebt u ´s nachts daadwerkelijk gehad? (Dit kan verschillend zijn van het aantal uren dat u in bed lag.) *

(…uren) vóór sociale isolatie EN (…uren) tijdens sociale isolatie

5. Hoe vaak hebt u slaapproblemen gehad omdat u…

786. 5a. Niet kon inslapen binnen de 30 minuten *

Mark only one oval per row.

	Nooit	Minder dan eenmaal per week	Een- of tweemaal per week	Driemaal of meer per week
Vóór sociale isolatie				
Tijdens sociale isolatie				

787. 5b. Wakker werd in het midden van de nacht of te vroeg ´s ochtends *

Mark only one oval per row.

	Nooit	Minder dan eenmaal per week	Een- of tweemaal per week	Driemaal of meer per week
Vóór sociale isolatie				
Tijdens sociale isolatie				

788. 5c. Moest opstaan om naar het toilet te gaan *

Mark only one oval per row.

	Nooit	Minder dan eenmaal per week	Een- of tweemaal per week	Driemaal of meer per week
Vóór sociale isolatie				
Tijdens sociale isolatie				

789. 5d. Niet vrij kon ademen, luid hoestte of snurkte *

Mark only one oval per row.

	Nooit	Minder dan eenmaal per week	Een- of tweemaal per week	Driemaal of meer per week
Vóór sociale isolatie				
Tijdens sociale isolatie				

790. 5e. Het te koud/ warm had *

Mark only one oval per row.

	Nooit	Minder dan eenmaal per week	Een- of tweemaal per week	Driemaal of meer per week
Vóór sociale isolatie				
Tijdens sociale isolatie				

791. 5f. Andere redenen voor slaapproblemen had (bijv. pijn, telefoongebruik, nare dromen etc.) *

Mark only one oval per row.

	Nooit	Minder dan eenmaal per week	Een- of tweemaal per week	Driemaal of meer per week
Vóór sociale isolatie	⬭	⬭	⬭	⬭
Tijdens sociale isolatie	⬭	⬭	⬭	⬭

792. 6. Hoe vaak hebt u geneesmiddelen genomen om u te helpen slapen (al dan niet op recept)? *

Mark only one oval per row.

	Nooit	Minder dan eenmaal per week	Een- of tweemaal per week	Driemaal of meer per week
Vóór sociale isolatie	⬭	⬭	⬭	⬭
Tijdens sociale isolatie	⬭	⬭	⬭	⬭

793. 7. Hoe vaak hebt u de afgelopen maand moeite gehad om wakker te blijven tijdens het rijden, eten of deelnemen aan sociale activiteiten? *

Mark only one oval per row.

	Nooit	Minder dan eenmaal per week	Een- of tweemaal per week	Driemaal of meer per week
Vóór sociale isolatie	⬭	⬭	⬭	⬭
Tijdens sociale isolatie	⬭	⬭	⬭	⬭

794. 8. Hoe moeilijk was het de afgelopen maand voor u om voldoende gemotiveerd te blijven om dingen gedaan te krijgen? *

Mark only one oval per row.

	Helemaal niet moeilijk	Een klein beetje moeilijk	Nogal moeilijk	Heel moeilijk
Vóór sociale isolatie	◯	◯	◯	◯
Tijdens sociale isolatie	◯	◯	◯	◯

795. 9. Hoe zou u de kwaliteit van uw slaap in het algemeen beoordelen? *

Mark only one oval per row.

	Heel goed	Redelijk goed	Redelijk slecht	Heel slecht
Vóór sociale isolatie	◯	◯	◯	◯
Tijdens sociale isolatie	◯	◯	◯	◯

Bijkomende vragen

796. Selecteer a.u.b. wat op u het meest van toepassing is. *

Mark only one oval.

- ◯ Ik verleen op dit moment onbetaalde zorg (mantelzorg) aan een oudere (bijv. een partner, ouders, ander familielid of buurman/-vrouw).
- ◯ Ik ontvang zelf mantelzorg (bijv. van partner of ander familielid etc.) en/ of professionele thuiszorg. *Skip to question 800*
- ◯ Ik verleen professionele zorg aan ouderen. *Skip to question 801*
- ◯ Geen *Skip to question 804*

Reden van zorg, woonsituatie en belasting

797. Wat is de reden van zorg? (meerdere antwoorden mogelijk) *

Check all that apply.

- ☐ Ouderdom
- ☐ Dementie of milde cognitieve beperking (MCI)

Other: ☐ ____________________

798. Welke woonsituatie is op u van toepassing? *

Mark only one oval.

- Ik woon in hetzelfde huis als degene voor wie ik zorg.
- Degene voor wie ik zorg woont niet bij mij in huis.

799. In welke mate ervaart u belasting/ stress als gevolg van uw zorgtaken? *

Mark only one oval per row.

	Geen tot zeer kleine mate	In kleine mate	In gemiddelde mate	In grote mate	In zeer grote mate
Vóór de COVID-19 maatregelen					
Tijdens de COVID-19 maatregelen					

Skip to question 804

Reden van zorg

800. Wat is de reden van zorg? (meerdere antwoorden mogelijk) *

Check all that apply.

- [ ] Ouderdom
- [ ] Dementie of milde cognitieve beperking (MCI)
- Other: [ ] ______________________

Skip to question 804

Zorgsetting, reden van zorg en belasting

801. Selecteer a.u.b. wat op u het meest van toepassing is. *

Mark only one oval.

- Ik verleen professionele thuiszorg aan ouderen.
- Ik verleen zorg in de intramurale ouderenzorg.

802. Wat is de reden van zorg? (meerdere antwoorden mogelijk) *

Check all that apply.

- [ ] Ouderdom
- [ ] Dementie of milde cognitieve beperking (MCI)

Other: [ ] ____________________

803. In welke mate ervaart u belasting/ stress als gevolg van uw zorgtaken? *

Mark only one oval per row.

	Geen tot zeer kleine mate	In kleine mate	In gemiddelde mate	In grote mate	In zeer grote mate
Vóór de COVID-19 maatregelen	◯	◯	◯	◯	◯
Tijdens de COVID-19 maatregelen	◯	◯	◯	◯	◯

Skip to question 804

Eenzaamheid

804. In welke mate voelt u zich eenzaam? *

Mark only one oval per row.

	Geen tot zeer kleine mate	In kleine mate	In gemiddelde mate	In grote mate	In zeer grote mate
Vóór de COVID-19 maatregelen	◯	◯	◯	◯	◯
Tijdens de COVID-19 maatregelen	◯	◯	◯	◯	◯

Gebruik van communicatietechnologie

805. Welke digitale communicatiemiddelen gebruikte u vóór de COVID-19 maatregelen om contact met anderen te houden en hoe tevreden was u over deze middelen? Voor personen die (mantel)zorg aan een oudere verlenen: richt u zich a.u.b. op de hulpmiddelen die u gebruikte om contact te houden met de persoon voor wie u zorgt. *

Mark only one oval per row.

	Zeer ontevreden	Ontevreden	Neutraal	Tevreden	Zeer tevreden	Niet gebruikt
Telefoon	⬭	⬭	⬭	⬭	⬭	⬭
Beeldbellen	⬭	⬭	⬭	⬭	⬭	⬭
Social Media	⬭	⬭	⬭	⬭	⬭	⬭
Online platforms voor (mantel)zorg	⬭	⬭	⬭	⬭	⬭	⬭
Messenger apps	⬭	⬭	⬭	⬭	⬭	⬭

806. Welke digitale communicatiemiddelen gebruikt u op dit moment om contact met anderen te houden en hoe tevreden bent u over deze middelen? Voor personen die (mantel)zorg aan een oudere verlenen: richt u zich a.u.b. op de hulpmiddelen die u nu gebruikt om veilig contact te houden met de persoon voor wie u zorgt. *

Mark only one oval per row.

	Zeer ontevreden	Ontevreden	Neutraal	Tevreden	Zeer tevreden	Niet gebruikt
Telefoon	⬭	⬭	⬭	⬭	⬭	⬭
Beeldbellen	⬭	⬭	⬭	⬭	⬭	⬭
Social Media	⬭	⬭	⬭	⬭	⬭	⬭
Online platforms voor (mantel)zorg	⬭	⬭	⬭	⬭	⬭	⬭
Messenger apps	⬭	⬭	⬭	⬭	⬭	⬭

807. Welk ander digitaal communicatiemiddel gebruikt u op dit moment nog meer? (Indien niet van toepassing, sla vraag over)

____

808. Hoe tevreden bent u over dit communicatiemiddel? (Indien niet van toepassing, sla vraag over)

Mark only one oval.

	1	2	3	4	5	
Zeer ontevreden	◯	◯	◯	◯	◯	Zeer tevreden

809. Wij vinden het erg belangrijk om mensen te kunnen betrekken bij het ontwikkelen en het verbeteren van technologie die ondersteuning kan bieden. Zouden wij u mogen benaderen voor toekomstig onderzoek? Zo ja, dan kunt u hieronder uw e-mailadres voor ons achterlaten (vrijwillig).

____

Επιδράσεις του οικιακού περιορισμού σε πολλές συμπεριφορές τρόπου ζωής κατά τη διάρκεια της επιδημίας COVID-19 (ECLB-COVID19)

Προστασία προσωπικών δεδομένων / Ασφάλεια

Η προστασία δεδομένων και η προστασία της ιδιωτικής ζωής είναι υψίστης σημασίας. Η παρούσα ανώνυμη διαδικτυακή έρευνα σχεδιάστηκε μόνο για ερευνητικούς σκοπούς. Στο ερευνητικό μας έργο δίνουμε ιδιαίτερη μέριμνα για την προστασία της ιδιωτικής ζωής και της ασφάλειας των δεδομένων και προσπαθούμε να προστατεύσουμε τα δεδομένα που συλλέγονται από τυχόν μη εξουσιοδοτημένη πρόσβαση τρίτων, λαμβάνοντας υπόψη τους πιο πρόσφατους κανονισμούς όσον αφορά την προστασία δεδομένων.
Συνεπώς, οι πληροφορίες που συλλέγονται στο ECLB-COVID19 διατηρούνται με αυστηρή εχεμύθεια. Κατά τη διαδικασία της ενημερωμένης συναίνεσης, οι συμμετέχοντες στην έρευνα είναι σίγουροι ότι τα δεδομένα που συλλέγονται θα χρησιμοποιηθούν μόνο για ερευνητικούς σκοπούς και δεν θα αποκαλυφθούν ούτε θα διατεθούν σε άλλους χωρίς τη συγκατάθεση του ατόμου. Ωστόσο, επειδή χρησιμοποιούμε τα Έντυπα Google, οι συμμετέχοντες πρέπει επίσης να αναγνωρίζουν την πολιτική απορρήτου της Google (https://policies.google.com/privacy?hl=el).
Πρέπει να είστε 18 ετών και άνω για να συμμετάσχετε σε αυτή τη μελέτη.

Συγκατάθεση Συμμετοχής

Συμπληρώνοντας αυτήν την έρευνα, συμφωνείτε να συμμετέχετε ανώνυμα σε αυτή τη μελέτη.

If you are using your smartphone to answer this questionnaire, please drag the cursor to see all the possible answers

810. Χώρα (πρώτο γράμμα κεφαλαίο) *

____

811. Πόλη *

____
